# Real-time mortality statistics during the COVID-19 pandemic: A proposal based on Spanish data, January–March, 2021

**DOI:** 10.3389/fpubh.2022.950469

**Published:** 2022-11-08

**Authors:** Juan Equiza-Goñi

**Affiliations:** Facultad de Ciencias Económicas y Empresariales, Universidad de Navarra, Pamplona, España

**Keywords:** public health surveillance, mortality, COVID-19, nowcasting, health communication

## Abstract

**Objectives:**

During the COVID-19 pandemic, surveillance systems worldwide underestimated mortality in real time due to longer death reporting lags. In Spain, the mortality monitor “MoMo” published downward biased excess mortality estimates daily. I study the correction of such bias using polynomial regressions in data from January to March 2021 for Spain and the Comunitat Valenciana, the region with the highest excess mortality.

**Methods:**

This adjustment for real-time statistics consisted of (1) estimating forthcoming revisions with polynomial regressions of past revisions, and (2) multiplying the daily-published excess mortality by these estimated revisions. The accuracy of the corrected estimates compared to the original was measured by contrasting their mean absolute errors (MAE) and root mean square errors (RMSE).

**Results:**

Applying quadratic and cubic regressions improved the first communication of cumulative mortality in Spain by 2–3%, on average, and the flow in registered deaths by 20%. However, for the Comunitat Valenciana, those corrections improved the first publications of the cumulative mortality by 36–45%, on average; their second publication, by 23–30%; and the third, by 15–21%. The flow of deaths registered each day improved by 62–63% on their first publication, by 19–36% on the second, and by 12–17% on the third.

**Conclusion:**

It is recommended that MoMo's estimates for excess mortality be corrected from the effect of death reporting lags by using polynomial regressions. This holds for the flows in each date and their cumulative sum, as well as national and regional data. These adjustments can be applied by surveillance systems in other countries.

## Introduction

MoMo is the monitoring system of daily mortality for all causes in Spain. It was developed in 2004 and belongs to the European network of surveillance systems EuroMOMO. Since January 2020, daily mortality data is collected by almost 4,000 computerized civil registries, including all regions and representing 93% of the Spanish population. Since the COVID-19 pandemic reached Spain, the National Center of Epidemiology—Institute of Health Carlos III (Centro Nacional de Epidemiología—Instituto de Salud Carlos III, CNE-ISCII) has been issuing reports (1) on the evolution of excess mortality registered by MoMo as well as publishing daily data online on the observed mortality (2). The logic behind the publication of this information is that the statistic for excess mortality is also a good way of estimating and communicating to the public the number of deaths caused by COVID-19 [see, for example, Cerda Thomas (3), or, more recently, Adam (4)].

According to MoMo, Spain witnessed the fourth episode of excess mortality between 4 January and 13 February^1^. Despite the absence of lockdowns during this period and the experience acquired in previous COVID-19 surges, the number of deaths published in the first quarter of 2021 was still influenced by the aggravated reporting lag documented during similar episodes. Equiza-Goñi (5, 6) showed that the statistics of cumulative excess mortality in Spain in March–May and September–December of 2020 were downward biased due to death reporting lags. Akhmetzhanov (7) and Rosenbaum et al. (8) documented the same problem in all-cause excess mortality figures in the United States^2^.

By contrast, González Morán et al. (9) and Leon et al. (10) stressed the need for real-time information to monitor the pandemic. Aroca (11) and Vestergaard and Mølbak (12) underlined the importance of effectively communicating it to the public. Naudé and Vinuesa (13) and Malecki et al. (14) summarized some of the challenges that this communication implied and provided some recommendations. Carvalho et al. (15) suggested that nowcasting techniques must be applied to Brazilian mortality data to achieve more up-to-date monitoring. Gutierrez et al. (16) provided empirical evidence of the effect of death reporting lags on individuals' beliefs and behavior in Mexico, and, consequently, on COVID-19 transmission.

In this line of research, Equiza-Goñi (5, 6) proposed and evaluated the performance of estimating polynomial regressions based on past revisions of real-time data to correct the effect of death reporting lags. Based on data from 2020, both studies found that quadratic and cubic regression-based corrections applied to mortality estimates in their first publication improved them notably, setting them closer to their definitive values. Although using cubic regressions brought the corrected mortality estimates slightly closer to their definitive values than quadratic regressions, those estimates that were adjusted using quadratic regressions were more robust to the presence of holidays in the sample (6). Extending previous work, the present study evaluates this methodology in new data from 2021 using not only aggregated national but also regional Spanish mortality estimates. Moreover, this is measured for the cumulative sum of deaths and, in a further step, for the flow reported for each date. As a novel measure, to gauge the full benefits of this methodology, estimates beyond their first publication were also corrected.

The main goal of this study is, thus, to evaluate the accuracy of extrapolating observed past revisions through polynomial regressions to correct the widespread negative bias derived from death reporting lags in real-time mortality estimates during the pandemic. For this purpose, I used a sample of Spanish data published by MoMo during the first quarter of 2021.

## Methodology

Equiza-Goñi (5) described and evaluated a methodology to correct the effect of death reporting lags in real-time excess mortality estimates published by MoMo. This method was followed in this study to adjust daily data published in January–March 2021. Note that MoMo uses the model in León-Gómez et al. (17) to define excess mortality as the difference between the observed and expected numbers of deaths. A similar procedure was followed by León-Gómez et al. (18) for all of Spain, or by Ochoa Sangrador et al. (19) for the case of the Spanish region “Comunidad Autónoma de Castilla y León”. However, there are other models for estimating excess mortality that could be applied, for example, Vanella et al. (20).

First, I obtained the excess mortality time series estimates^3^ for all of Spain and each of its autonomous regions or “Comunidades Autónomas” (CC.AA.) that were published daily by MoMo in the period 11 January to 31 March 2021. Note that, for each date of this period, MoMo published updated estimates for the complete daily history of mortality observed since 2015. I excluded publications before 2021 because these were not provided by MoMo between 5 and 10 January 2021. Given that I use both aggregated data for Spain and their CC.AA, my work is aligned with studies on previous episodes of excess mortality in the current pandemic using both national data [Fouillet et al. (22); Vestergaard et al. (23); regarding the first “wave”, and Nørgaard et al. (24); Grabowski et al. (25); in the context of the second] and regional level data [Morfeld et al. (26); Modig et al. (27); or, more recently, Konstantinoudis et al. (28)].

Second, based on the data released between 11 January and 31 March, I computed the intensity of daily revisions for the time series of excess mortality accumulated since 8 December 2020^4^. Revisions for each date of the time series are calculated as the ratio between the estimates published 1 day and the corresponding ones reported for the same dates the day before. For example, on 14 January, the excess mortality accumulated up to 13 January was published at 2,790 deaths. By contrast, the sum of deaths for that period had been 2,213 as of 13 January, which is the first release extending the series of mortality up to that date. This means that on 14 January, the cumulative excess mortality estimate that was firstly published the day before was revised by 126% (or, simply, 1.26). Thus 1.26 is an example of an observed revision performed 1 day after (the first) publication. The data published on 14 January also reported that the cumulative excess mortality up to 12 January came to 2,736 deaths. However, the data as of 13 January provided a cumulative excess mortality for that period of 2,319 deaths. Since the excess mortality for that period was first published on 12 January, I conclude that between 13 and 14 January-−2 days after the first publication—the sum of deaths for that period was revised by 118%. Thus, 1.18 is an example of a revision performed 2 days after (first) publication.

Third, I corrected the excess mortality estimates in the sample: both the series of cumulative excess deaths, and the flows of deaths registered daily. The corrections of the former were extrapolations into the future of the past, already observed, revisions. I started correcting publications on 18 January, and thus had the releases dated 11–18 January as the initial sample of revisions, and thereafter, it was gradually extended as an increasing window. The extrapolations were based on quadratic and cubic regressions estimated with the data on revisions that I had computed^5^. The corrected flows, however, were the first differences of the cumulative excess mortality estimates already adjusted using polynomial regressions. I also imposed in all cases that the adjustments (i.e., the extrapolated revisions) had to be ratios greater or equal to one (i.e., published estimates can only be revised upwards). Moreover, the vertex or inflection point of the fitted curve would necessarily take the value of 1 in the vertical axis; and only the decreasing part of the curve is considered relevant.

**Figure 1:**
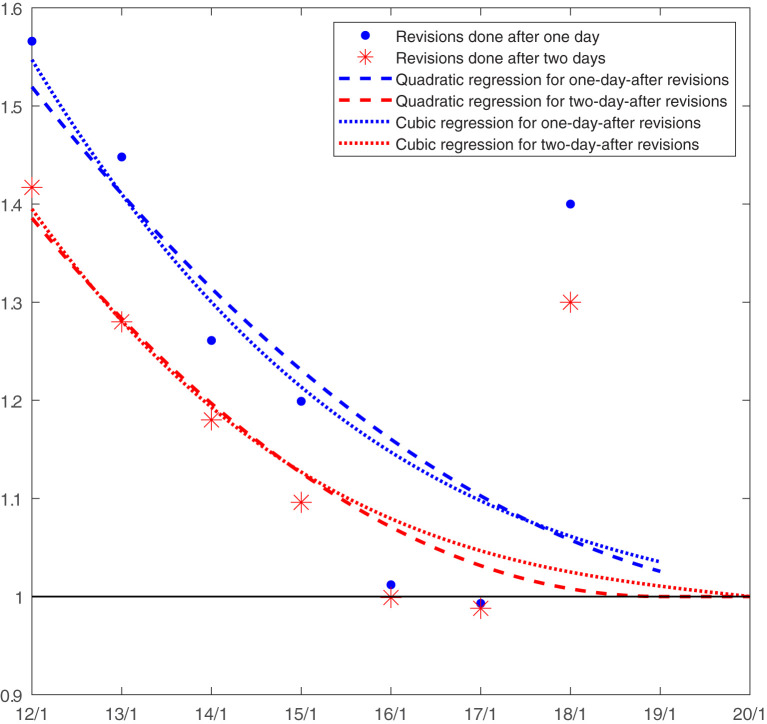
Estimation of the 1-day-after and 2-day-after publication revisions for the 18 January release. Source: author own calculations.

Figure 1 shows an example: the estimated 1-day and 2-day after-publication adjustments for the 18 January data release. The thick blue dots show the revisions performed between 12 and 18 January of cumulative mortality estimates 1 day after their first publication (i.e., of those first published on 11–17 January). The red asterisks show, instead, the revisions performed on 12–18 January of cumulative mortality estimates 2 days after their first publication (i.e., of those first published on 10–16 January). Note that the values corresponding to 14 January are both mentioned in the previous paragraph: 1.26 and 1.18. The blue and red dashed lines are the quadratic regressions estimated to predict the 1-day and 2-day after-publication revisions, respectively. The revision carried out on the excess of mortality first published on 18 January, the day after is predicted by extrapolating the regression based on observed 1-day after revisions to 19 January obtaining 1.026. Similarly, the revision 2 days after the first publication for the 18 January estimate is forecasted by extrapolating the regression based on observed 2-day after-revisions on 20 January. Given that the fitted curve has its vertex on 19 January, the revision forecasted for 20 January is necessarily equal to 1.00. The blue and red dotted lines are the predicted revisions based on cubic regressions, instead: 1.035 for 19 January as a 1-day after-revision, and 1.00 for 20 January as a 2-day after-revision^6^. Similarly, if estimated revisions 3, 4, 5… up to 14 days after, and multiplied all of them by the cumulative excess mortality first published up to 18 January, I would obtain my corrected estimate of excess mortality for that date and release.

Finally, I compared the accuracy of the real-time estimates (either published by MoMo or already corrected using this methodology) with respect to their definitive values. For this purpose, I used the well-known criteria: mean absolute error (MAE) and (square) root of the mean squared error (RMSE), both frequently used measures of the difference between predicted and observed values. I compared the published and corrected estimates not only in their first publication but also in the posterior ones.

## Results

**Figure 2:**
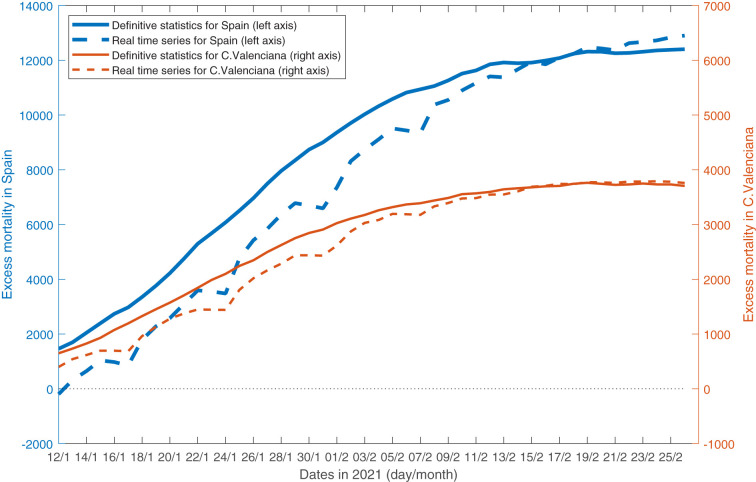
Cumulative excess mortality in Spain and the *C. valenciana* on 12 January to 26 February 2021. Source: datasets retrieved daily by the author in that period from https://momo.isciii.es/panel_momo/.

Figure 2 shows the time series published between 12 January and 31 March for the cumulative excess mortality in Spain (blue solid line) as well as the first estimates published each day during that period (dotted orange line)^7^. Given that the former (i.e., the revised series published at the end of the first quarter of 2021) became stable and close to the latter (i.e., the estimates published in real time) by 19 February, Figure 2 shows only the subsample ending on 26 February 2021. It can be seen that these real-time estimates were downward biased because of death reporting lags. This delay implied that the excess mortality estimated daily was, on average, 91% of its definitive value (assuming the latter to be the series published at the end of the first quarter).

Using polynomial regressions to correct Spanish real-time excess mortality estimates in their first release resulted in values that represented, on average, 92% of the already revised estimates published at the end of the quarter. In particular, the first column in Table 1 shows that the mean absolute error (MAE) of the corrected estimates divided by the MAE of the original estimates released in their first publication (“After 0 days”) is equal to 0.98. Similarly, the ratio between the (square) root mean squared error (RMSE) of corrected and original firstly published estimates is 0.98. Summing up, correcting these first publications using quadratic regressions implied an improvement in these by measures close to 2% (with respect to the original, non-corrected, estimates). Below, in the same column, I show that cubic regressions also implied an improvement of about 3%.

**Table 1:** Measures of accuracy for corrected mortality estimates on 18 January to 26 February 2021.

**Panel A. Data for Spain**								
	**Cumulative estimates**				**Daily flows**			
**After:**	**0 days**	**1 day**	**2 days**	**3 days**	**0 days**	**1 day**	**2 days**	**3 days**
**Quadratic regressions**
MAE	0.98	0.99	0.99	0.99	1.09	1.11	1.06	0.97
RMSE	0.98	0.99	0.99	0.99	0.91	1.01	1.01	1.01
Improvement summary	0.02	0.01	0.01	0.01	0	−0.06	−0.03	0.01
**Cubic regressions**
MAE	0.97	0.99	0.98	0.99	0.72	0.86	0.86	0.92
RMSE	0.97	0.99	0.99	0.99	0.88	1	1	0.99
Improvement summary	0.03	0.01	0.01	0.01	0.20	0.07	0.07	0.04
**Panel B. Data for Comunitat Valenciana**								
	**Cumulative estimates**				**Daily flows**			
**After:**	**0 days**	**1 day**	**2 days**	**3 days**	**0 days**	**1 day**	**2 days**	**3 days**
**Quadratic regressions**
MAE	0.63	0.76	0.83	0.87	0.39	0.61	0.80	0.89
RMSE	0.64	0.79	0.87	1	0.38	0.67	0.85	0.93
Improvement summary	0.36	0.23	0.15	0.11	0.62	0.36	0.17	0.09
**Cubic regressions**
MAE	0.50	0.66	0.76	0.85	0.36	0.80	0.87	0.89
RMSE	0.60	0.74	0.82	1	0.39	0.81	0.88	0.91
Improvement summary	0.45	0.30	0.21	0.12	0.63	0.19	0.12	0.10

Labels ***MAE*** and ***RMSE*** denote the ratio of each of these measures for the case of corrected estimates (in the numerator) and original, non-adjusted, estimates (in the denominator). The ***Improvement summary*** is equal to 1 minus the average of the ratios for MAE and RMSE. The validity of the corrections based on quadratic and cubic regressions was measured **after 0 days** of their publication (that is, at the time of their first publication), as well as **after 1, 2, and 3 days**. Both **cumulative estimates** and **daily flows** of the registered number of deaths were corrected.

The next three columns in Table 1 show that the corrections based on both regressions (quadratic and cubic) improved by about 1% the official statistics that had been already revised once, twice, and three times (days) after their first publication. In the next columns, on the right, I show the ratios concerning the number of deaths registered each day (the flows) in contrast to the cumulative excess mortality already discussed. We can see that the cubic model corrections applied to the first published estimates brought them 20% closer, on average, to their definitive values; and 7% closer 2 or 3 days after publication. The corrections based on quadratic regressions did not improve real time statistics.

The aggregated data for Spain, however, hides a large heterogeneity between regions or CC.AA. Table 2 shows for each region the total number of deaths registered during the first quarter of 2021 and the difference with respect to the expected mortality, i.e., the excess mortality. In the third column, I show the ratio between this excess mortality and the total number of deaths. For example, 10% of total mortality registered for Spain as a whole between 1 January and 31 March 2021 was actually in excess with respect to the usual figure. However, this percentage is lower in many CC.AA. (e.g., Asturias, with 7%; or Galicia, with 2%) and it is even negative in a few cases (e.g., Canarias, −6%, or Navarra, −5%). The Comunitat Valenciana, in contrast, suffered excess mortality of about 22% of its total number of registered deaths.

**Table 2:** Total number of deaths and registered excess mortality from 1 January to 31 March 2021.

	**Total number of deaths**	**Excess mortality**	**Ratio excess/total mortality**
Andalucía	23,374	3,109	0.13
Aragón	3,356	282	0.08
Asturias	3,830	254	0.07
Baleares	2,367	80,5	0.03
Canarias	4,359	−245	−0.06
Cantabria	1,161	−164	−0.14
Castilla—La Mancha	5,830	599	0.10
Castilla y León	6,533	272	0.04
Cataluña	19,877	1,764	0.09
Comunitat Valenciana	16,117	3,466	0.22
Extremadura	3,347	468	0.14
Galicia	9,060	185	0.02
Comunidad de Madrid	12,902	1,696	0.13
Murcia	3,744	492	0.13
Navarra	1,472	−72	−0.05
País Vasco	5,924	578	0.01
La Rioja	749	118	0.16
SPAIN	124,340	12,443	0.10

Names of the CC.AA. stated as they appear in the MoMo Reports but Ceuta and Melilla were omitted. Source: data retrieved by the author on 1 April 2021, from https://momo.isciii.es/panel_momo/.

Figure 2 also shows the cumulative excess mortality in the Comunitat Valenciana between 12 January and 26 February 2021. Note that there is also a difference between the historical series (thin solid blue line) and the estimates published in real time (thin dotted orange line). In fact, on average, the preliminary statistics on excess mortality published daily represented around 94% of their definitive values, that is, those already revised and published at the end of the first quarter of 2021.

Table 2 shows that the Comunitat Valenciana was the “autonomous region” with the highest cumulative excess mortality as a share of its total number of deaths in the first quarter of 2021. The second half of Table 1 reports the benefits of applying quadratic and cubic regressions to correct the mortality estimates published by MoMo for this region^8^. Interestingly, the statistics published first in real time for the regional cumulative excess mortality became 36 and 45% closer to their definitive values after they were corrected using 2nd- and 3rd-grade polynomials. Previously, it was mentioned that the first publication of these mortality estimates was, on average, 94% of their revised values; now, after being corrected they represented, on average, 98–99% of their definitive values.

The three columns that follow in the second half of Table 1 show that both types of regressions generate improvements of about 23–30% and 15–21% in real-time estimates that had already been revised once and twice (1 and 2 days) after their first publication, respectively^9^. In the columns on the right reporting ratios for daily mortality flows, we can see that the corrections brought their originally published values 62–63% closer to their definitive values. In the following days, when these estimates had already been revised once or twice, the corrections yielded improvements of about 19–36% and 12–17%, respectively. Summing up, the regression-based adjustments refined very significantly the publications of the statistics for this region at least up to their third revision.

## Discussion

Table 1 shows that using quadratic and cubic models to correct the effect of reporting lags in excess mortality estimates at the national level brought them slightly closer to their definitive values. By comparison, Equiza-Goñi (5, 6) reported improvements of about 18–25 and 6–13% in the periods 15 April to 25 May 25 and 1 September to 25 December 2020, respectively. This different performance could be explained by the smaller share of the most recent excess mortality on the total number of registered deaths (10%) compared to the two previous excess mortality episodes (39 and 15%, respectively). Moreover, Table 2 documents a ratio of excess mortality relative to total mortality much higher for the Comunitat Valenciana than for the rest of the CC.AA. According to the second half of Table 1, the real-time statistics for this autonomous region did become notably closer to their definitive values after the polynomial corrections had been applied.

Therefore, Table 1 shows that the polynomial corrections used in national-level data seem to be much more relevant in regional data, plausibly because there is a relatively important excess mortality. At least, that is clearly the case for the Comunitat Valenciana. Moreover, Table 1 also shows that the correction of the negative bias caused by reporting lags has persistent effects, being beneficial even when the real-time estimates have been revised repeatedly. In addition, it can be observed that most of the improvement in the cumulative excess mortality estimates is due to bringing the data of the last, most recent, flows of registered deaths closer to their definitive values. In fact, MoMo states in the files documenting their real-time online dataset or “dashboard” that the number of deaths reported in the most recent days previous to the date of publication might be susceptible to modification in the days that followed (29). My work shows that those revisions can be effectively forecasted days in advance using polynomial regressions, and thus bringing MoMo's preliminary estimates of these flows closer to their actual values. This information can be especially useful to achieve updated monitoring of the pandemic, thus complementing the other commonly used indicators, such as the daily number of cases and hospital or ICU admission rates.

Finally, Table 1 reports that the corrections based on cubic regressions generally improved the statistics published in real time more than by corrections using quadratic regressions. This is not surprising given that the second-order polynomial is embedded in the cubic model (i.e., a restricted polynomial). Equiza-Goñi (6) found, however, the opposite result and claimed that it was due to the presence of several holidays between 1 September and 25 December 2021. The problem was that holidays implied longer reporting lags for reasons unrelated to the number of deaths and the time since their first publication. The higher flexibility of the cubic model compared to the quadratic regression resulted in the estimates from Equiza-Goñi (6) being more sensitive to these outliers (i.e., unusual revisions for common values of excess mortality and days past since publication). Given that in the timespan covered in this study, only March 19 was a holiday in the Comunitat Valenciana, the higher performance of cubic over quadratic regressions is aligned with the discussion in Equiza-Goñi (6). Thus, the best corrections would result from using quadratic regressions in the presence of a relevant number of holidays in the sample, while using cubic regressions in their absence.

In summary, it is recommended that MoMo's daily statistics on excess mortality be corrected by applying revisions predicted by polynomial models that have already been estimated with data from past revisions. In this way, using quadratic and cubic regressions helps mitigate the negative bias observed in real-time estimates due to death reporting lags. Other recent studies (30, 31) consider this delay as one of the main obstacles to the correct interpretation of COVID-19 data. Sarnaglia et al. (32) or Guglielmi et al. (33), for example, suggest other methodologies to reduce the problems generated by reporting lags in counting the number of infectious cases. This work suggests that, in the pandemic, the estimates of mortality that are currently reported by surveillance systems worldwide, and by MoMo and EuroMOMO in particular, could be complemented by publishing also these adjusted measures. In such a manner, these systems will not simply acknowledge that in periods of extraordinary mortality reporting lags could worsen but, in addition, provide public health practitioners estimates in which foreseeable revisions have already been implemented. Also, the real-time communication to the public of mortality statistics that are not systematically downward biased will raise awareness about public health risks and incentivize more responsible behavior.

Moreover, as noted, estimates corrected using the cubic model are generally closer to their definitive values if the estimating sample does not include holidays; otherwise, using quadratic estimates seems a more robust option. Therefore, a mixed strategy combining both quadratic and cubic extrapolations would be desirable, depending on whether holidays constitute a significant part of the sample period. All corrected estimates are nonetheless preliminary and, thus, should be subjected to a critical reading (34). For instance, even though this study adds further evidence of the improvements achieved through polynomial extrapolations, adjusted real-time estimates would still be remarkably revised. Moreover, at times, the corrections recommended by this research would result in inflated mortality estimates that, arguably, would generate an unnecessary alarm. Overall, the advantages of more accurate mortality real-time data could easily compensate for some possible drawbacks. Further work could also test these methodological recommendations and their benefits in sample periods of excess mortality due to extreme temperatures or the flu.

## Data availability statement

The original contributions presented in the study are included in the article/supplementary material, further inquiries can be directed to the corresponding author.

## Author contributions

Author contributed to the article and approved the submitted version.

## Conflict of interest

The author declares that the research was conducted in the absence of any commercial or financial relationships that could be construed as a potential conflict of interest.

## Publisher's note

All claims expressed in this article are solely those of the authors and do not necessarily represent those of their affiliated organizations, or those of the publisher, the editors and the reviewers. Any product that may be evaluated in this article, or claim that may be made by its manufacturer, is not guaranteed or endorsed by the publisher.

## Footnotes

^1^The media have often named that period as “the third wave”, thus ignoring the second episode that happened in the summer of 2020 and, fortunately, translated only into a moderate mortality.

^2^Surveillance systems in the U.S.A. and Europe share many characteristics, specifically, they all rely on largely computerized processes. However, U.S. and EuroMOMO data are published weekly while the Spanish MoMo system is reporting data daily.

^3^Balak et al. (21) studies the estimation and prediction of the cumulative number of COVID-19 cases.

^4^The chosen starting date of the accumulation period is flexible and implies only slight differences in the results.

^5^In line with Equiza-Goñi (5, 6), using polynomials of order 1 yielded little or no improvement, thus this article focuses on presenting only the benefits of using quadratic and cubic regressions.

^6^In the case of 2-day after-revisions, the fitted cubic curve has precisely an inflexion point on 20 January.

^7^The accumulation period for excess mortality shown in Figure 2 starts on 1 January. When applying polynomials, I used figures of excess mortality accumulated since 8 December, 2020, to avoid the negative sum resulting from figures published up to 18 January.

^8^I normalized the size of the cumulative excess mortality of the Comunitat Valenciana to be equal to that of Spain by accumulating it from an earlier date than 8 December. Thus, the different size of excess mortality does have an impact on the results; that is, all differences are due to the relatively bigger revisions (longer reporting lags) observed in the region compared to Spain.

^9^Not shown in Table 2, these corrections generated improvements of about 11–13% and 8–9% in real time estimates that had already been revised three and four times after their first publication, respectively.
